# Protective Activity of the CnaBE3 Domain Conserved among *Staphylococcus aureus* Sdr Proteins

**DOI:** 10.1371/journal.pone.0074718

**Published:** 2013-09-17

**Authors:** Marco Becherelli, Prachi Prachi, Elisa Viciani, Massimiliano Biagini, Luigi Fiaschi, Emiliano Chiarot, Sarah Nosari, Cecilia Brettoni, Sara Marchi, Marco Biancucci, Maria Rita Fontana, Francesca Montagnani, Fabio Bagnoli, Michèle A. Barocchi, Andrea G. O. Manetti

**Affiliations:** 1 Novartis Vaccines and Diagnostics, Siena, Italy; 2 Department of Medical Biotechnologies, University of Siena, Siena, Italy; University of California, San Francisco, United States of America

## Abstract

*Staphylococcus aureus* is an opportunistic pathogen, commensal of the human skin and nares, but also responsible for invasive nosocomial as well as community acquired infections. *Staphylococcus aureus* adheres to the host tissues by means of surface adhesins, such as SdrC, SdrD, and SdrE proteins. The Sdr family of proteins together with a functional A domain, contain respectively two, three or five repeated sequences called B motifs which comprise the CnaB domains. SdrD and SdrE proteins were reported to be protective in animal models against invasive diseases or lethal challenge with human clinical *S. aureus* isolates. In this study we identified a 126 amino acid sequence containing a CnaB domain, conserved among the three Sdr proteins. The three fragments defined here as CnaBC2, D5 and E3 domains even though belonging to phylogenetically distinct strains, displayed high sequence similarity. Based on the sequence conservation data, we selected the CnaBE3 domain for further analysis and characterization. Polyclonal antibodies raised against the recombinant CnaBE3 domain recognized SdrE, SdrC and SdrD proteins of different *S. aureus* lineages. Moreover, we demonstrated that the CnaBE3 domain was expressed *in vivo* during *S. aureus* infections, and that immunization of this domain alone significantly reduces the bacterial load in mice challenged with *S. aureus*. Furthermore, we show that the reduction of bacteria by CnaBE3 vaccination is due to functional antibodies. Finally, we demonstrated that the region of the SdrE protein containing the CnaBE3 domain was resistant to trypsin digestion, a characteristic often associated with the presence of an isopeptide bond.

## Introduction

*Staphylococcus aureus* is a Gram positive opportunistic pathogen associated with asymptomatic colonization of the skin and mucosal surfaces. This microorganism is responsible for infections in humans and animals, ranging from mild localized impetigo and cellulitis, to life threatening systemic infections such as endocarditis, osteomyelitis, toxic shock syndrome and gastroenteritis [1]. *S. aureus* is one of the most important causes of nosocomial (catheters and implants) and community acquired infections [2,3], and being a reservoir of multiple antibiotic resistance genes, favors the rapid spread of drug resistant isolates, such as methicillin resistant strains (MRSA) [4,5]. Overall the incidence of staphylococcal diseases has increased over the past ten years [6], supporting the importance of developing a vaccine that can prevent life-threatening infections [7,8].

As many other microbial pathogens, *S. aureus* adheres to the host tissues by means of MSCRAMMs (microbial surface components recognizing adhesive matrix molecules), which recognize fibronectin, fibrinogen, collagen, and heparin related polysaccharides and are responsible for the initial contact with host cells [9]. Sdr (derived from the repetition of amino acid serine –S- and aspartic acid –D-) are MSCRAMM proteins involved in adherence to epithelial cells [10,11], and structurally related to a family of cell wall anchored proteins known as ClfA and ClfB (clumping factor A and B) [12].

**Figure 1:**
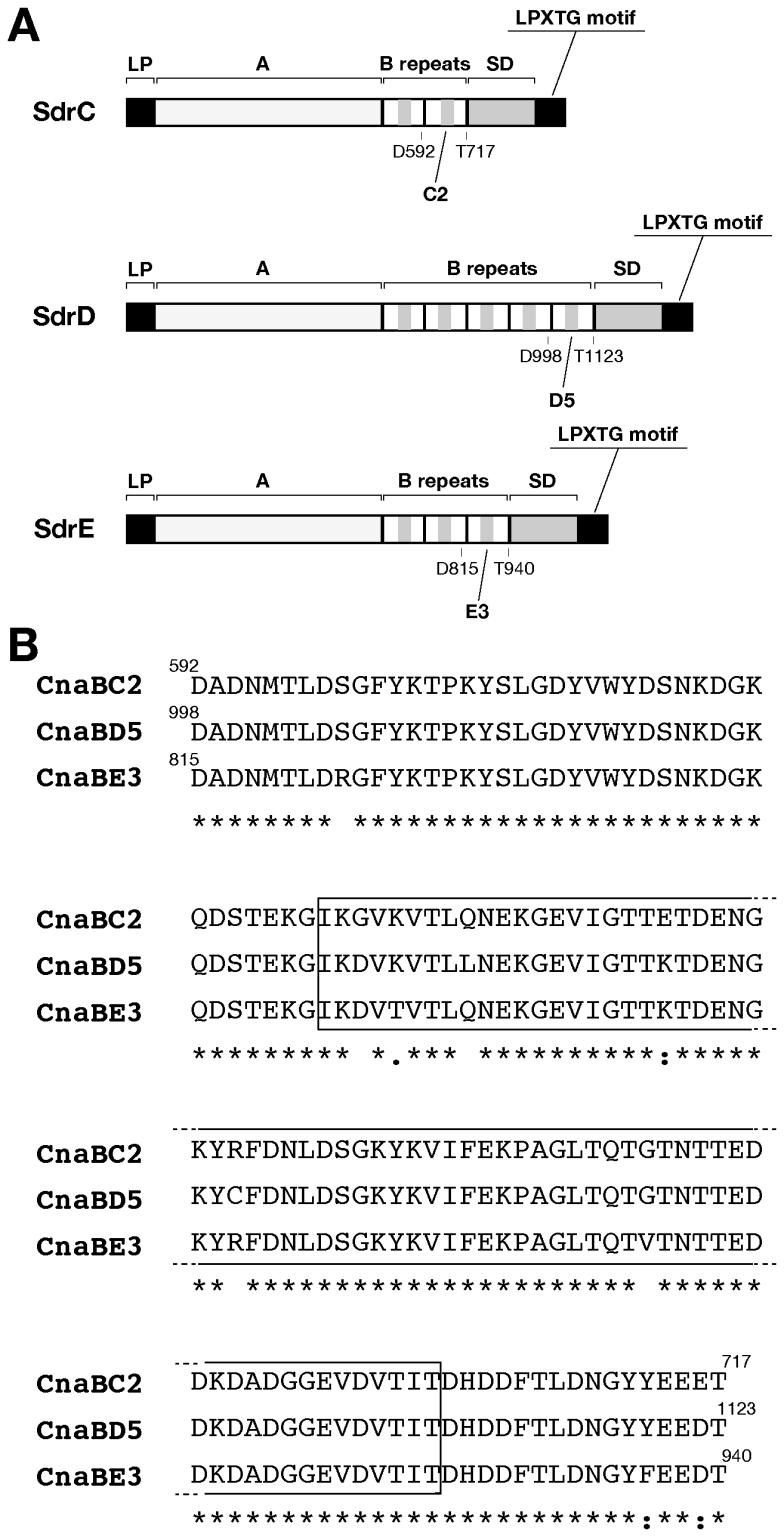
Schematic representation of Sdr proteins and amino acid sequence of CnaBC2 CnaBD5 and CnaBE3 domains. A) Schematic representation of Sdr proteins. A putative leader peptide (LP) sequence and an LPXTG motif are depicted in black. The A domain is reported in light gray, whereas B repeats (two, three, or five, for SdrC, SdrE, and SdrD, respectively) are shown in white, and contain putative CnaB domains shown in dark gray. Finally, at the C-terminus, the SD repeat domain is depicted in dark gray. In addition, boundaries of CnaBC2, CnaBD5 and CnaBE3 domains are reported. B) CnaBC2, CnaBD5 and CnaBE3 domain amino acid sequences are aligned. Identical residues are highlighted, and putative CnaB domains are encompassed by a black box.

The *sdr* locus encodes three proteins, SdrC, SdrD, and SdrE, each of them composed of a putative leader peptide sequence at the N-terminus, followed by an A domain and by two, three, or five 110-113 residue repeated sequences (for SdrC, SdrE, and SdrD, respectively), called B repeats containing the CnaB domains. These domains are hypothesized to function as spacers which regulate the distance between the interactive A domain and the surface of the bacteria. The C-terminal region of the Sdr proteins contain the SD repeat domain composed of 132-170 S-D residues, followed by an LPXTG motif [12] (see Figure 1A).

In 2006, Stranger-Jones and colleagues demonstrated that immunization with a four antigen combination containing SdrD and SdrE proteins was able to generate significant protective immunity against invasive disease or lethal challenge with human clinical *S. aureus* isolates in a kidney abscess and/or in a lethal mouse animal model [7].

In this paper, following sequence analysis of the three Sdr proteins of the *S. aureus* Newman strain, we identified a 126 amino acid sequence conserved among the three Sdr proteins, with a sequence identity comprised between 94 and 97%. Since the identified fragments encompassed the second, the third and fifth B repeat of SdrC, SdrD, and SdrE respectively, we termed them as CnaBC2, D5 and E3 domains. We report significant sequence similarity among Sdr full length proteins especially among their CnaB domains, even when belonging to phylogenetically distinct strains. Based on the conservation data we selected CnaBE3 domain for further analysis. Polyclonal antibodies raised against the recombinant CnaBE3 domain were able to recognize all the three full length Sdr proteins belonging to the same panel of *S. aureus* lineages. Moreover, ELISA experiments performed using patient or healthy donor sera showed that a specific immune response against CnaBE3 domain was raised during *S. aureus* infections. Furthermore, mice immunized with the CnaBE3 domain showed a significant reduction in bacterial load, when challenged intravenously with either Newman strain (SdrE positive strain) or the NCTC8325 strain (SdrE negative, but SdrC and SdrD positive strain). These data strongly suggest that the sequence similarity shared by CnaB domains E3, C2 and D5 was sufficient to promote cross protection in a mouse model of kidney abscess formation. Finally, we showed that mice vaccinated with CnaBE3 vaccination produced functional anti-CnaBE3 antibodies, able to mediate the killing of the *S. aureus* Newman bacteria by differentiated HL-60 cells. Interestingly, the region of the SdrE protein containing the CnaBE3 domain was resistant to trypsin digestion, a characteristic often associated with the presence of an isopeptide bond. However, the analysis of two different constructs containing CnaBE3 domain has not revealed thus far the presence of intramolecular isopeptide bonds.

## Results

### The CnaBE3 domain sequence is highly conserved among phylogenetically distinct strains

**Figure 2:**
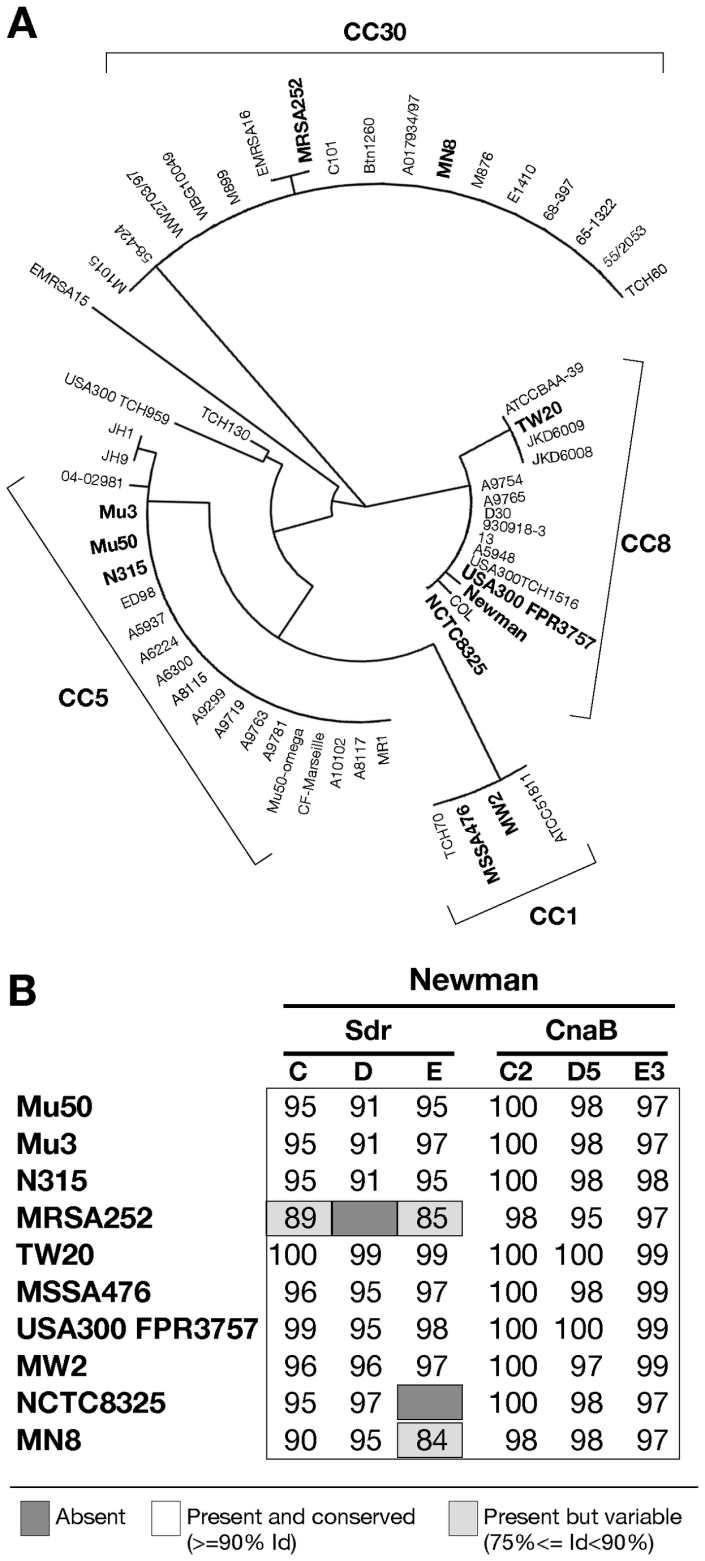
CnaBE3 domain sequence is highly conserved among phylogenetically distinct strains. A) The depicted phylogenetic tree was obtained using the Sequence Types (ST) of a panel of 59 epidemiologically relevant *S. aureus* strains. Clonal complexes 1, 5, 8 and 30 are highlighted. The eleven bacterial strains selected for conservation analysis are in bold. B) The percentages of amino acid sequence identity obtained from the comparison of Sdr proteins and the CnaBC2, D5 and E3 domain amino acid sequences of Newman strain to those of an epidemiologically relevant panel of *S. aureus* strains are reported. Dark gray color means that proteins are absent, whereas white color means present and conserved with an identity percentage ≥ 90, and light gray color means present but variable with an identity percentage ≥ 75 and ≤ 89, on at least 75% of the amino acid sequence.

As previously reported, SdrE and SdrD proteins are known to be protective against *S. aureus* infection in a mouse model of kidney abscess formation [7]. Therefore, we hypothesized that a shared component of the two related proteins could be responsible for the protection; hence, sequence analysis of the three Sdr proteins of the *S. aureus* Newman strain was performed. We identified a 126 amino acid conserved sequence present in all the three Sdr proteins. The amino acid identity ranged between 94 and 97%, whereas the sequence identity obtained by comparing the three full length proteins never exceeded 43% (Table 1). The identified polypeptides, located adjacently to the SD region, contained a CnaB domain, and encompassed the second, the third and fifth B repeat of SdrC, SdrE, and SdrD respectively (Figure 1 A and B). Therefore we decided to name these fragments respectively as CnaBC2, D5 and E3 domains. Comparison of the amino acid sequence of the Sdr proteins and the CnaBC2, D5 and E3 domains of the Newman strain to a panel of clinical *S. aureus* isolates, showed high sequence similarity and conservation both among Sdr full length proteins and more specifically among their CnaB domains, even when belonging to phylogenetically distinct strains (Figure 2A). In fact, when considering CnaB domains, the percent identity was never below 95%. Remarkably, even in strains lacking *sdrD* (MRSA252) or *sdrE* genes (NCTC8325) the comparison with Newman CnaB domains, revealed a high percentage of amino acid sequence identity, due to the similarity of the lacking CnaB domains (CnaBD5 or CnaBE3) with those still present (e.g. CnaBC2) in these two genomes (Figure 2B).

**Table 1:** Amino acid identity between Sdr proteins sequences and between CnaBC2, CnaBD5 and CnaBE3 domain sequences.

	**% AA sequence identity**
**SdrC vs SdrD**	**34**
**SdrC vs SdrE**	**36**
**SdrE vs SdrD**	**43**
**CnaBC2 vs CnaBD5**	**97**
**CnaBC2 vs CnaBE3**	**94**
**CnaBE3 vs CnaBD5**	**94**

Amino acid sequence identity was calculated on at least 75% of the complete sequence both among Sdr full length proteins and among CnaBC2, CnaBE3 and CnaBD5 domains of the *S. aureus* Newman strain and expressed in percent.

We decided to further characterize the CnaBE3 domain, because SdrE is a reported virulence factor in invasive *S. aureus* infections [13], and it was found to be protective against *S. aureus* challenge in mice [7].

### Anti-CnaBE3 Domain Antibodies Recognize the Three Sdr Full Length Proteins

**Figure 3:**
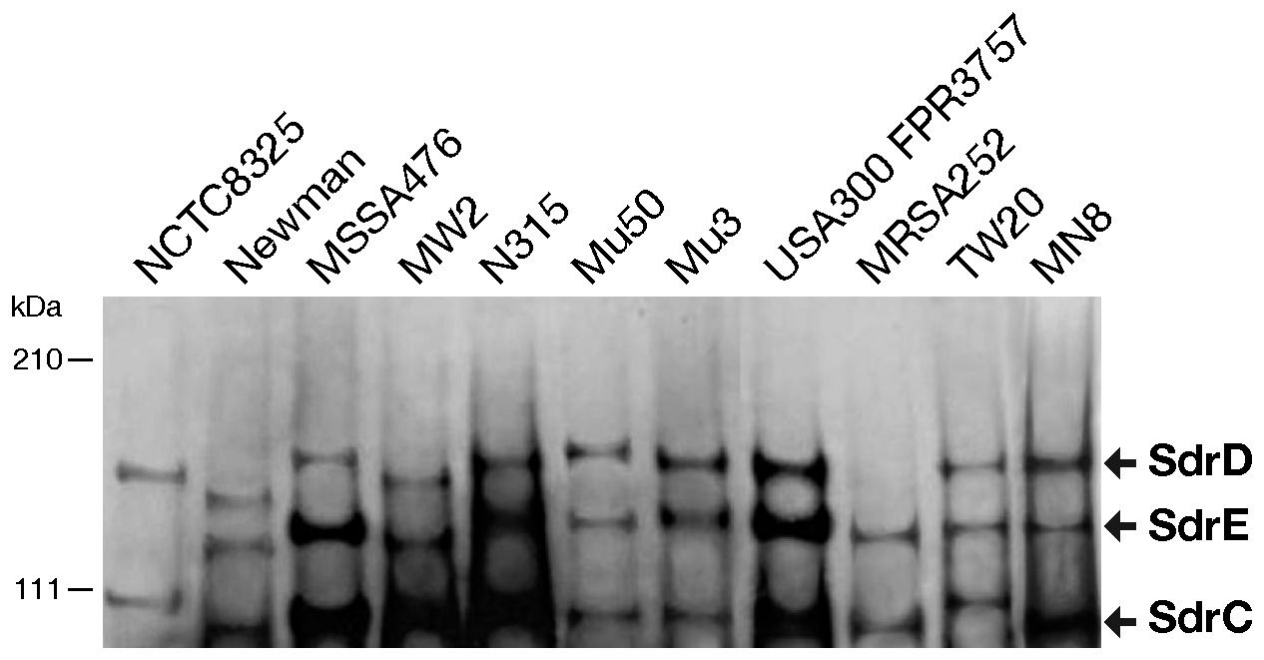
Anti-CnaBE3 domain antibodies recognize all the three Sdr full length proteins. Western blot analysis of bacterial cell wall extracts from NCTC8325, Newman, MSSA476, MW2, N315, Mu50, Mu3, USA300 FPR3757, MRSA252, TW20, and MN8 *S. aureus* strains blotted with anti-CnaBE3 domain antibodies. The Sdr proteins of each strain are highlighted.

Having described the high sequence similarity and conservation of CnaBE3 domain throughout a panel representative of the phylogenetic distance among *S. aureus* strains, we investigated whether polyclonal antibodies raised against the CnaBE3 domain were able to detect the three Sdr proteins in the same panel of *S. aureus* strains. As shown in Figure 3, the anti-CnaBE3 polyclonal antibodies recognized all the three Sdr proteins in all the protein extracts. As expected, neither protein SdrD in the *sdrD* negative strain MRSA252, nor protein SdrE in the *sdrE* negative strain NCTC8325 was detected. These data demonstrated that anti-CnaBE3 domain antibodies are able to recognize the SdrC and SdrD proteins, even when they belong to evolutionarily distinct strains.

### The CnaBE3 domain is recognized by sera of *S. aureus* infected patients

**Figure 4:**
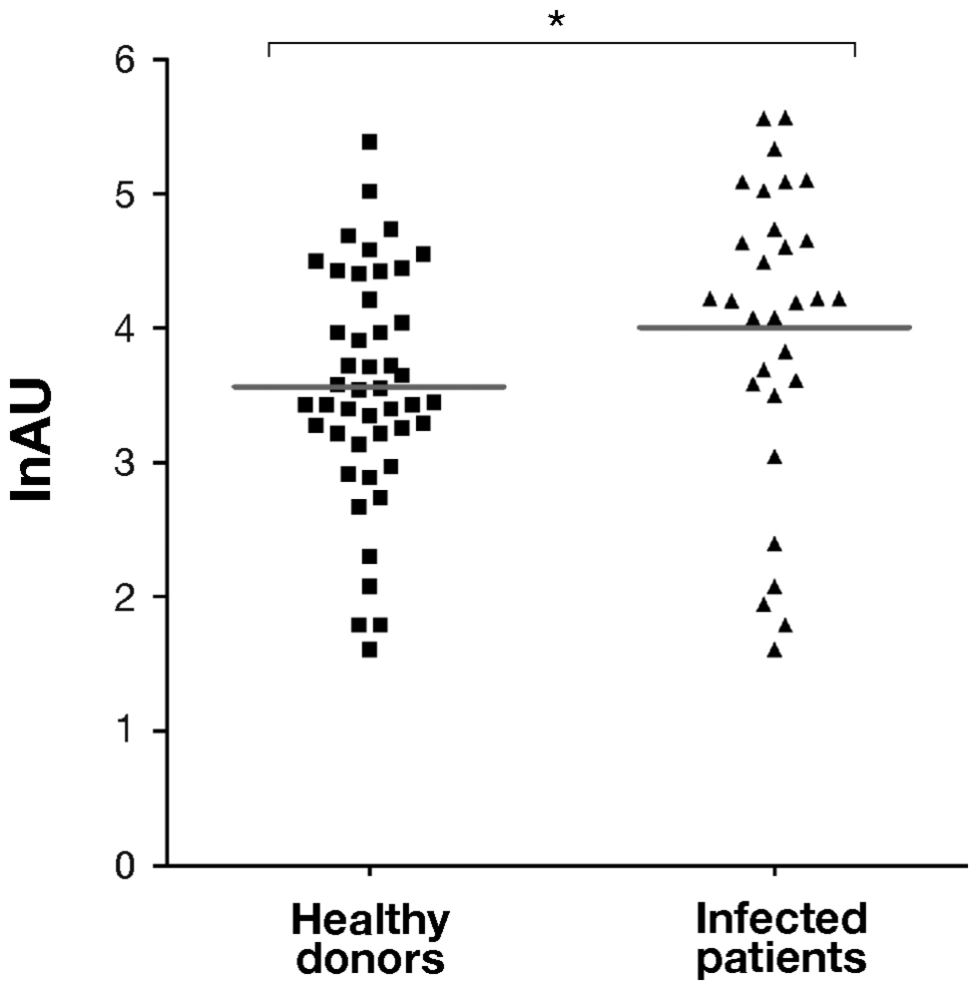
CnaBE3 domain is recognized by sera of *S. aureus* infected patients. The immune reactivity of a panel of 30 human sera collected from *S. aureus* infected patients was tested against the CnaBE3 domain by ELISA assay. A panel of 46 sera collected from healthy donors was used as control. Anti-CnaBE3 IgG titers of sera collected from the patients were significantly higher than those of sera collected from healthy donors. Each dot represents a single serum, and geometric means are reported. Statistical analysis was performed with a Mann–Whitney U test. Values are expressed in lnAU (natural logarithm of Arbitrary Units), for the calculation method see the material and method section (*p≤0.05).

To investigate whether CnaBE3 domain is expressed during a staphylococcal infection, a panel of 30 human sera collected from patients affected by different *S. aureus* diseases, such as pyodermitis, impetigo, pneumonia, endocarditis, arthritis and osteomyelitis, was tested against the CnaBE3 domain by an ELISA assay. Additionally a panel of 46 sera collected from healthy donors was used as control. As shown in Figure 4, anti-CnaBE3 IgG titers of sera collected from the patients were significantly higher than those of sera collected from healthy donors (*p*≤0.05). These data suggested that, even though up to 80% of the healthy population is a permanent (20%) or intermittent (60%) carrier of *S. aureus*, a specific immune response against CnaBE3 fragment was indeed raised during *S. aureus* infections.

### The CnaBE3 domain promotes significant bacterial load reduction in mice challenged with Newman strain and with NCTC8325 strain defective for *sdrE* gene

**Figure 5:**
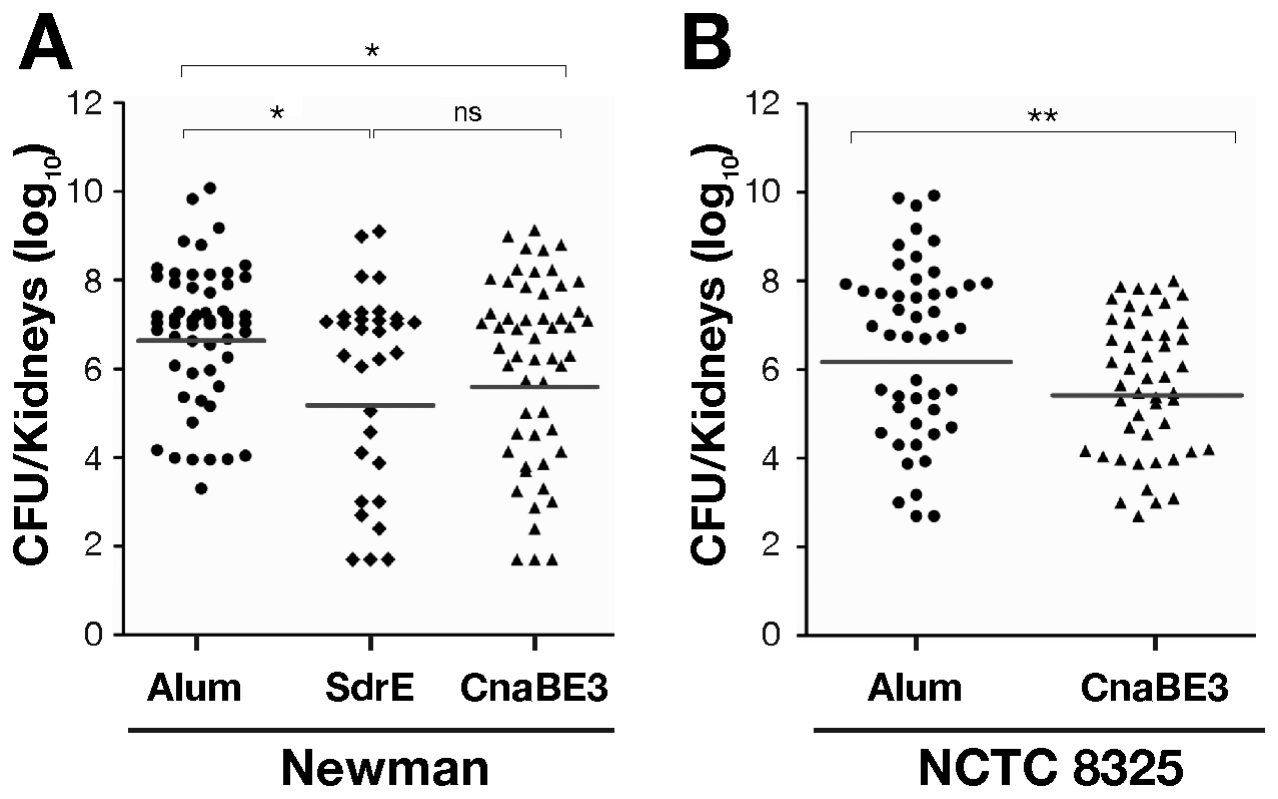
CnaBE3 domain promotes significant bacterial load reduction in mice challenged with Newman strain and with NCTC8325 strain defective for sdrE gene. Immunized mice were intravenously infected with either *Staphylococcus aureus* Newman (N = 54, 4 independent experiments) or NCTC8325 (sdrE negative) strains (N = 48, 3 independent experiments). A) Both, mice immunized with CnaBE3 domain or SdrE protein showed a significant reduction in bacterial load in kidneys when infected with *S. aureus* Newman strain if compared to adjuvant alone immunized mice used as negative control. B) Mice immunized with CnaBE3 domain show a significant reduction in bacterial load when challenged with NCTC8325 strain as compared to adjuvant alone immunized animals. Each dot represents a single mouse, and geometric means are reported. Statistical analysis was performed using a Mann–Whitney U test (*p≤0.05, **p≤0.01, ns means not significant).

The protective efficacy of the CnaBE3 domain was assessed in a mouse model of kidney abscess formation [7]. As reported in Figure 5A, immunization with the full length SdrE protein or the CnaBE3 domain reduced the bacterial load (as measured by CFU’s) in the kidneys of one logarithm, as compared to the negative control group (alum 4.27 x 10^6^ CFUs, SdrE 1.48 x 10^5^ CFUs, CnaBE3 3.89 x 10^5^ CFUs). Notably, no differences were observed between the bacterial load reductions obtained using either the CnaBE3 domain or the full length protein, suggesting that the bacterial CFU reduction observed in this latter group could be mainly due to the presence of an immune response raised against the CnaBE3 polypeptide. To further confirm that this domain was sufficient to induce a significant (*p*≤0.05) reduction in bacterial load and to evaluate the cross protection activity of CnaBE3 domain against SdrC or SdrD proteins, mice immunized with CnaBE3 domain were challenged using the *S. aureus* strain NCTC8325, which is naturally defective for the *sdrE* gene. As shown in panel B of Figure 5, mice immunization with CnaBE3 domain caused a significant CFU reduction in bacterial load in kidneys (2.63 x 10^5^ CFUs), when compared to mice immunized with Alum alone (1.48 x 10^6^ CFUs) (*p*≤0.01).

### Polyclonal antibodies specific for the CnaBE3 domain mediate killing of *S. aureus*

**Figure 6:**
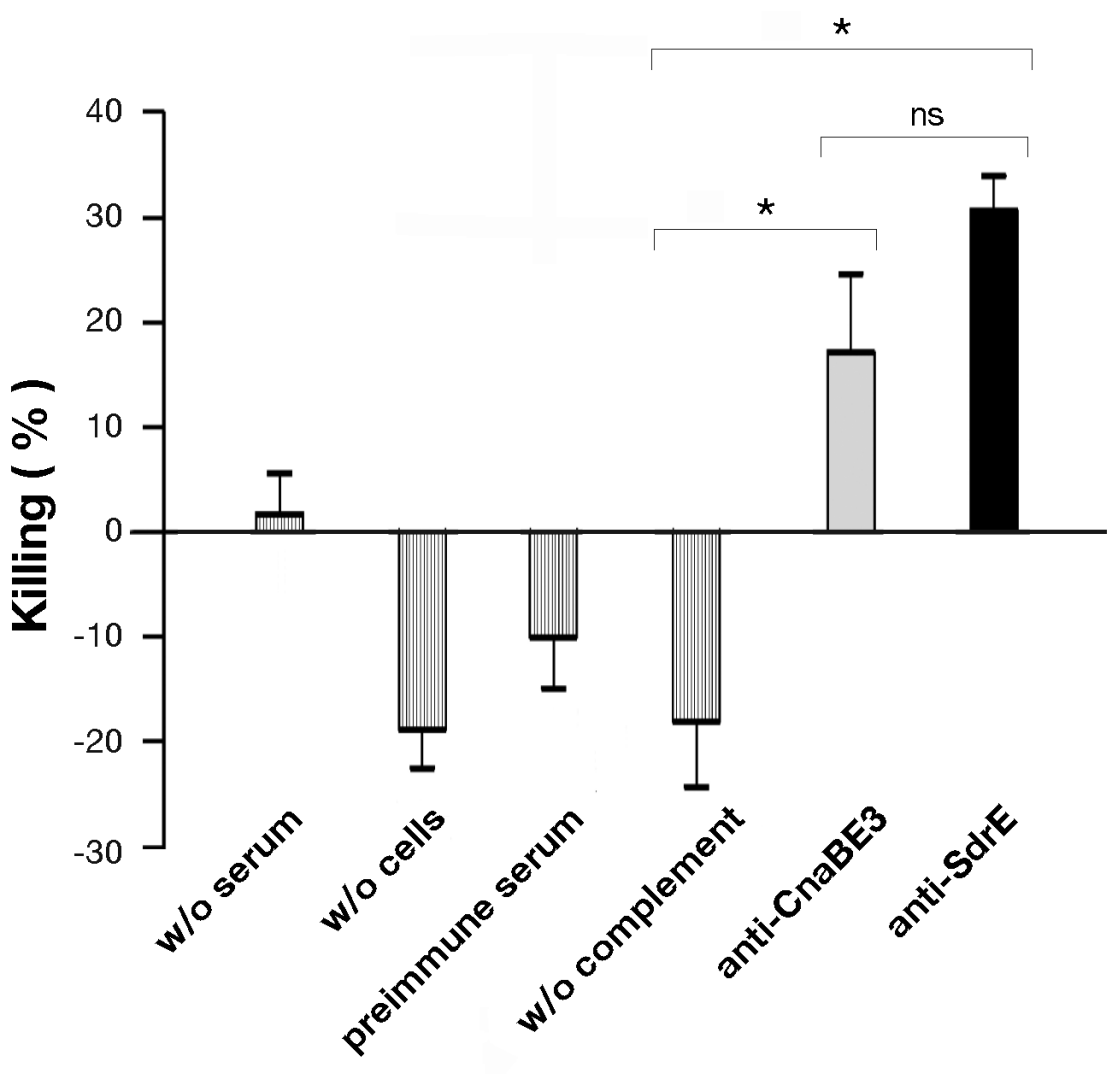
Antibodies against CnaBE3 and SdrE mediate opsonophagocytic killing of *S. aureus*. Sera of mice immunized either with CnaBE3 domain or with SdrE full length protein at dilution of 1:500, rabbit complement, human promyelocytic leukemia cells HL-60, and the *S. aureus* strain Newman were incubated for 1 h and plated on TSA for CFU counting. No bacterial killing was observed in the absence of serum, HL-60 cells, complement, or in presence of control serum (preimmune serum), showing the specificity of both CnaBE3 and SdrE antisera in mediating opsonophagocytic killing of the pathogen. Error bars represent standard deviation. Statistical analysis was performed by paired t test (*p≤0.05, ns means not significant). For calculation of the killing percentage see the material and method section.

To evaluate whether decrease in bacterial load by CnaBE3 immunization was associated with functional antibodies, we performed an *in vitro* opsonophagocytic killing assay (OPK). Sera samples from CnaBE3 and SdrE immunized mice were characterized for their ability to mediate killing of *Staphylococci* by differentiated HL-60 cells in the presence of rabbit complement. HL-60 cells killed 20% of Newman cells in the presence of anti-CnaBE3 serum and 30% in the presence of anti-SdrE serum, whereas no killing of the Newman strain was observed in the presence of the preimmune serum or in absence of either complement or cells (Figure 6). Of note, there was no difference observed between the percentages of OPK activity obtained using either the CnaBE3 domain or the full length protein, suggesting again that the killing of Newman cells mediated by the SdrE antiserum, could be mainly due to the CnaBE3 antibodies.

### The SdrE protein containing the CnaBE3 repeat is resistant to trypsin digestion

**Figure 7:**
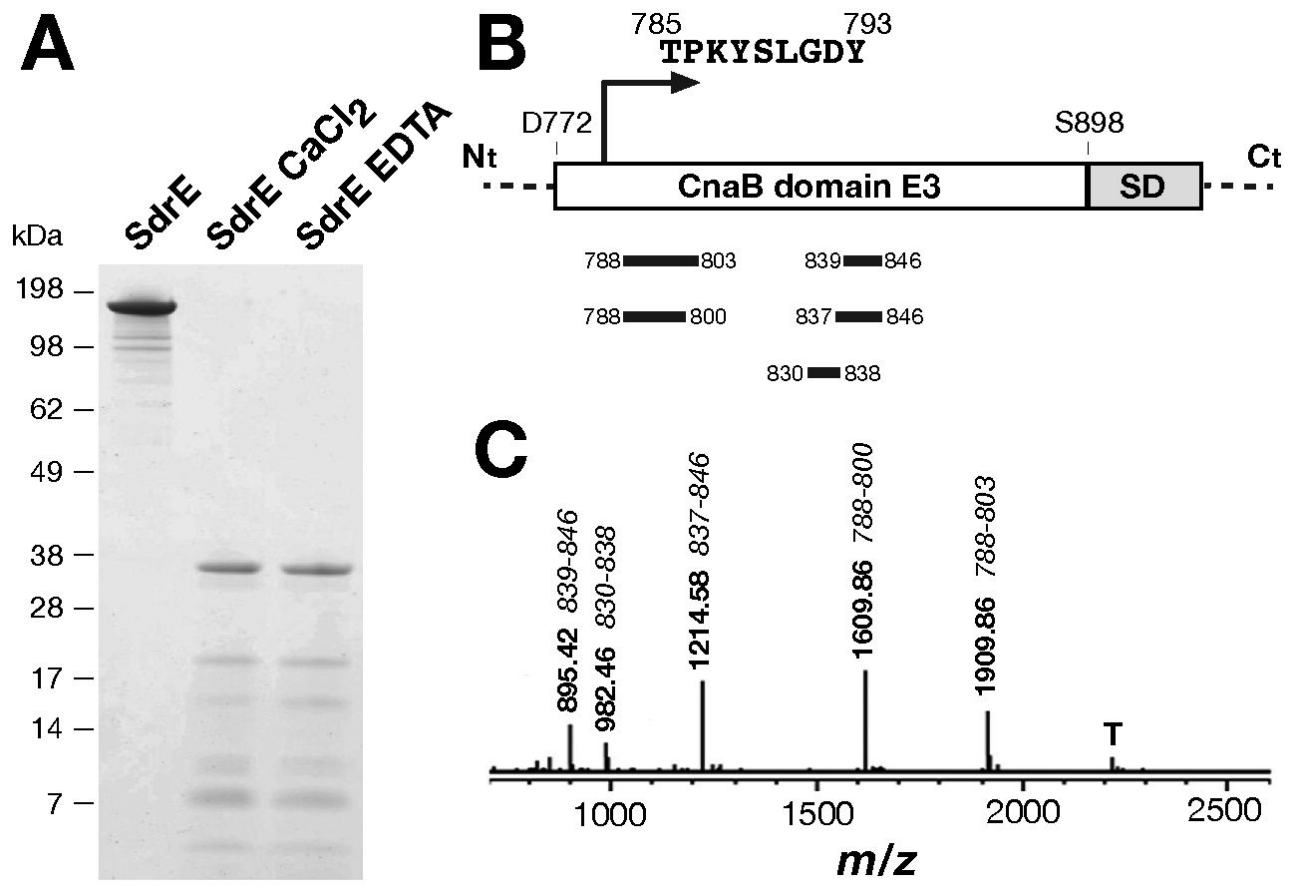
Trypsin digestion of full length SdrE protein and sequence analysis of the 37 kDa resistant fragment. A) SDS-PAGE analysis of an overnight trypsin digestion of the full length SdrE protein dialyzed either in 1 mM CaCl_2_ or in 1 mM EDTA. B) The C-terminal region of the full length SdrE protein is schematically depicted. The N-terminal sequence of the 37kDa resistant fragment obtained by Edman degradation (TPKYSLGDY) is shown, together with the peptides derived by trypsin digestion (black bars), and identified by analyzing the MALDI-TOF MS spectrum reported in panel C. “T” indicates trypsin autocatalytic fragment.

To further characterize the CnaBE3 domain as a putative vaccine candidate, we investigated its biochemical and structural properties. The CnaB domains of Gram positive pilus proteins contain intramolecular isopeptide bond, which confers resistance to enzymatic digestion [14]. Since Differential Scanning Fluorimetry (DSF) experiments demonstrated that calcium stabilizes SdrE protein (Figure S1), to rule out a possible influence of calcium in resistance to trypsin digestion, SdrE protein was dialyzed against CaCl_2_ or EDTA, and then subjected to trypsin digestion. As shown in Figure 7A, the overnight trypsin digestion resulted in one major band of 37 kDa in both conditions. The deduced N-terminal sequence of the trypsin resistant fragment was T-P-K-Y-S-L-G-D-Y-V corresponding to amino acid 785-793 of the recombinant protein SdrE and to amino acid 14-23 of the CnaBE3 domain (Figures 7B and 1B). Moreover, peptide mass fingerprint of the trypsin resistant fragment encompassed the region of the SdrE protein containing the CnaBE3 domain (peptides identified starting from amino acid 788 to amino acid 846 of the recombinant SdrE protein) (Figure 7B and 7C). The data are in agreement with the results from Edman degradation and with the apparent molecular weight observed in SDS-PAGE. In fact, no tryptic cleavage sites (K, R) are present in the SD repeat domain, which is located in the C-terminal part of the protein, immediately downstream to the CnaBE3 domain (Figure 7B). Therefore, we hypothesized that the CnaBE3 domain contains isopeptide bonds conferring resistance to trypsin digestion. Intact mass spectrometry analysis of the CnaBE3 did not demonstrate the loss of ~ 17 Da, as expected in the presence of an isopeptide bond (data not shown). These data indicated that the CnaBE3 domain does not assume a conformation compatible with the establishment of an isopeptide bond, without ruling out the hypothesis that this bond may occur in the full length protein.

## Discussion

Several MSCRAMMs have been thus far identified [9,15,16], among them the *Staphylococcus aureus* collagen-binding Cna, which contains a B region, that acts as a structural protein to expose the functional A region outside the bacterial cell. This B region is composed of a variable number of repeated sequences called CnaB domains, and displays an inverse IgG like fold [17]. Many CnaB-type proteins have been characterized in a number of bacterial species. In 1998, Josefsson and colleagues identified *S. aureus* Sdr proteins [12], which are involved in adherence to epithelial cells [10,11], contain putative CnaB domains [12], and have been reported to be protective against *S. aureus* infection [7].

In this report, we demonstrated that a newly identified polypeptide of 126 amino acids, shared by the three Sdr proteins and containing a CnaB domain, was important in inducing a significant (*p*≤0.05 and *p*≤0.01) bacterial load reduction in animal model of *S. aureus* infection. A sequence conservation analysis showed low sequence identity among Sdr full length proteins of the *S. aureus* Newman strain (max 43% identity), while the sequence identity of their CnaB domains (CnaBC2, CnaBD5 and CnaBE3) ranged between 94 and 97%. Accordingly, the B repeats adjacent to the SD region of each Sdr protein were reported to share a high sequence identity [12]. Interestingly, the B motifs of the Sdr proteins display some sequence similarity to the B repeats of the collagen binding protein Cna of *S. aureus* [12]. Of note, the sequence of the third B repeat of the SdrE protein shows similarities with subdomain D1 of the CnaB1 domain of the *S. aureus* protein Cna, suggesting a putative extension of the sequence of CnaB domain of the third B repeat [17] (Figure S2 panel A and B). Of interest, this 126 amino acid polypeptide presents sequence similarities with CnaB domains identified in other species such as *Streptococcus agalactiae*, *pyogenes* and *pneumoniae*. These data led us to analyze the conservation of the three CnaB domains in a panel of eleven *S. aureus* strains, on the basis of their Multi Locus Sequence Typing (MLST) [18-21]. The conservation analysis of Sdr proteins and their CnaB domains showed that CnaBE3, CnaBD5 and CnaBC2 domains were highly conserved among the selected *S. aureus* strains, even with the absence of *sdrE* in strain NCTC8325 and of *sdrD* in strain MRSA252 (see Figure 2B). Several types of insertion and deletion have been found both in *sdrC* and sdr*D* genes of bovine, ovine and human isolates suggesting horizontal gene transfer as a means for variability. However, deletions of the region containing C2 or D5 domains and mutations in the *sdrE* gene have not yet been described [22], suggesting a stability of these Sdr regions. Interestingly, Xue and coworkers reported an higher conservation of *sdrE* gene (respect to *sdrC* or *sdrD*) in the two main phylogenetic clusters of *S. aureus sdr* genes analyzed [22]. Of these three genes, only *sdrC* is present in all *S. aureus* strains currently tested [13,23]. Due to the phylogenetic stability of the SdrE protein [22], we selected CnaBE3 domain for further analysis.

Analysis of protein expression in the eleven strains revealed that anti-CnaBE3 antibodies were able to detect the three Sdr proteins in the cell surface extracts of each strain tested; suggesting that these antibodies could recognize *S. aureus* strains lacking the *sdrE* gene. The three *sdr* genes were demonstrated to be transcribed as three separate transcripts regulated by environmental changes. It is reported that magnesium and calcium ions enhanced the expression of all *sdr* genes, especially sdrC, at different growth phases [24]. Of note, each B motif of the Sdr proteins contains a consensus Ca^2+^ binding EF-hand loop (see Figure S2), reported to be functional in the five B repeats of SdrD protein, which promote a compact conformation of the protein [25]. Our results demonstrate clearly that the full length protein SdrE is stabilized by the uptake of Ca^2+^ ions.

Data from ELISA provide evidence that sera from patients with *S. aureus* infections recognize the CnaBE3 domain when compared with sera from healthy donors, suggesting that CnaBE3 is expressed during *S. aureus* infections. It is noteworthy that sera collected from a healthy population recognize the CnaBE3 domain. This is probably due to the fact that up to 80% of the healthy population has been a carrier of *S. aureus* [26,27]. Interestingly, Dryla and colleagues reported a significantly higher IgG level against SdrD protein in the sera from acute phase *S. aureus* infected patients, when compared with healthy donors [28]. This suggests that SdrD was expressed early during disease and recognized by the immune system [28].

Both SdrD and SdrE full length proteins have been previously reported as protective antigens in combination with two other vaccine candidates [7]. Furthermore, it has recently been shown that SdrD protein is instrumental in the process of abscess formation [29]. Our results indicate that, in a renal abscess formation mouse model, immunization with CnaBE3 domain provides a significant reduction of the bacterial load, when challenged with *S. aureus*, with the same efficacy as the full length SdrE protein.

Moreover, immunization with the CnaBE3 domain, whose sequence is conserved and widespread in *S. aureus*, showed similar results when challenged with strain NCTC8325 (defective for the *sdrE* gene), implying that CnaBE3 antibodies could recognize SdrC and/or SdrD proteins. The OPK data showed that anti-CnaBE3 antibodies mediated opsonophagocytosis of *S. aureus*, suggesting that the observed bacterial load reduction was, at least partially, antibody mediated, as reported for antibodies raised against other *S. aureus* antigens [30,31].

The CnaB domains identified in Gram positive pilus proteins have been described to contain intramolecular isopeptide bonds [14,32-38], conferring resistance to enzymatic digestion; however, isopeptide bonds within putative CnaB domains of Sdr proteins have yet to be reported. Of note, the SdrE protein was identified only when subject to harsh preparation in an exoproteome analysis; this feature has previously been observed for pilus proteins of *Streptococcus* species (Nathalie Norais, personal communication). Our results show that no isopeptide bond was identified in the CnaBE3 domain; nevertheless, it is not possible to rule out the hypothesis that the formation of an isopeptide bond may occur in the SdrE full length protein, expressed as a different construct or *in vivo* by *S. aureus* [39,40].

Despite the introduction of new antibiotics, mortality, morbidity, and cost from invasive *S. aureus* infections remain high [41], supporting the rational to develop a vaccine to prevent diseases caused by *S. aureus* [7,8,41,42]. Optimal vaccine antigens should be immunogenic, expressed by the majority of clinical *S. aureus* isolates, and should elicit antibodies that promote opsonophagocytic killing [42].

Our data suggest the CnaBE3 domain as a possible vaccine candidate, supported by the high conservation of the CnaBE3 sequence and by its immunogenicity and expression during *S. aureus* infections. Functional domains, which interact with host ligands, are generally considered as promising vaccine candidates. However, the most variable protein domains are the putative functional regions, which undergo selective pressure in order to circumvent the host immune system reaction [43]. On the contrary, domains such as CnaBE3 are well conserved, due to their importance in maintaining the structure of the protein. As previously shown by other groups, antigen combinations can confer greater protection than single antigens in vaccine formulations [7,44]. Hence, CnaBE3 would have to be included in a multicomponent vaccine, even as a fusion protein, along with other comprising toxoids and antigens that target the complexity and different stages of the pathogenesis of *S. aureus* diseases.

## Materials and Methods

### Bacterial strains, recombinant protein and antiserum

*Staphylococcus aureus* strains Newman, NCTC8325, MSSA476, MW2, N315, Mu50, Mu3, MRSA252 and MN8 (NRS112) were obtained from Professor Schneewind at University of Chicago, USA300 FPR3757 from Professor Binh Diep at University of California of San Francisco and TW20 from Professor Jonathan Edgeworth at Divisional Medical Director for Ambulatory Patient Care Guy’s and St. Thomas’ Hospital Foundation Trust. Bacteria were maintained on TSA agar plates supplemented with 5% defibrinated sheep blood and grown in liquid cultures at 37°C in Tryptic Soy Broth (TSB). *E. coli* BL21(DE3)-T1^R^ and One Shot Mach1-T1^R^ chemically competent cells (Invitrogen) were grown in LB medium. Selective medium contained 100 µg/ml ampicillin. The gene encoding SdrE protein was amplified from Newman strain, without predicted signal peptide coding sequences, using primers: sdrEF 5’-CTGTACTTCCAGGGCGCTGAAAACACTAGTACAGAA AATGCAAAACAAG and sdrER 5’-AATTAAGTCGCGTTATGCTTTTGCTTTATTG TGATGGTCTTTAGTAG-3’. CnaBE3 and CnaBE3 short form fragments were amplified from Newman genomic DNA, using primers: cnaBE3F 5’-GAAGGA GATATACATATGGATGCAGATAATATGACATTAGAC-3’ and cnaBE3R 5’-GTGGT GGTGGTGGTGTGTATCTTCTTCGAAGTATCCGTT-3’ and pp15E3sF 5’-CTG TACTTCCAGGGCAAATACAGTTTAGGTGATTATGTTTGGT-3’ and pp15E3sR 5’-AATTAAGTCGCGTTATGTATCTTCTTCGAAGTATCCGTTATCA-3’. All the obtained amplicons were cloned with the PIPE (Polymerase Incomplete Primer Extension) system [45] into the *E. coli* plasmid vector pET-15b+ (Novagen) to produce 6XHis fusions. The recombinant bacteria were propagated with the EnPresso Tablet Cultivation Set (BioSilta) [46,47]. The recombinant proteins were purified by affinity chromatography on His-Trap HP columns (GE Healthcare), and specific antisera were obtained by immunizing CD1 mice with purified recombinant proteins.

### Sequence analysis

Phylogenetic tree was obtained with Phylogeny.fr web service [48,49], using “One Click” mode and default options, and the Sequence Types (ST) of a panel of 59 epidemiologically relevant, publicly available *S. aureus* strains (http://sareus.mlst.net/). Eleven bacterial strains belonging to four out of ten dominant clonal complexes of *S. aureus* currently available on NCBI, were selected for further analysis [50]. All amino acid sequences used in this study were downloaded from bacterial genomes maintained at the NCBI site (http://www.ncbi.nlm.nih.gov/genome/genomes/154). First we used FASTA and BLAST software included in the Wisconsin package version 10.0, Genetics Computer Group (GCG) to identify and compare Sdr proteins and CnaB domains. The sequences of Sdr proteins and CnaB domains from the different strains were then aligned with clustalW [51], using default options and manually inspected for ambiguously aligned regions. Alignments reported in Table 1, Figure 1, and in Figure S2 were performed using clustal Omega [52,53], using default options and manually inspected for ambiguously aligned regions.

### Western Blot

Bacterial cell wall extracts were obtained as described previously [10]. *S. aureus* mid exponential phase cultures were grown in TSB supplemented with 5 mM CaCl_2_ to OD_600_ = 0.6. Cells were washed once in PBS and resuspended in 100 µl Lysis Buffer (50 mM Tris-HCl, 20 mM MgCl_2_, pH 7.5) supplemented with 30% raffinose, and 1X EDTA free protease inhibitors cocktail (Roche). 8 µg of lysostaphin (Sigma-Aldrich) were applied to the mix for 10 min at 37°C to harvest cell wall proteins. Extracts were centrifuged at 6,000 *xg* for 20 min at 4°C, and the supernatants containing cell wall extracts were boiled for 10 min with NuPAGE LDS Sample Buffer and NuPAGE Sample Reducing Agent (Life Technologies) and separated in 3-8% NuPAGE Tris-Acetate Gels (Invitrogen). Electrophoretically separated protein samples were transferred onto nitrocellulose membranes (Invitrogen) with iBlot gel transfer device (Invitrogen). Membranes were blocked, 2 hr on a shaker incubator (25°C, 700 rpm), in 10% skim milk (Bio-Rad) in TPBS (0.05% Tween 20 in PBS, pH 7.4). After three washes in TPBS, mouse polyclonal anti-CnaBE3 diluted 1:1,000 in 1% skim milk in TPBS was added and membranes were incubated for 1 hr (25°C, 700 rpm). Membranes were washed three times in TPBS and polyclonal rabbit anti-mouse immunoglobulins-HRP (Dako) diluted 1:5,000 in 1% skim milk in TPBS was added. After 1 hr (25°C, 700 rpm), membranes were washed three times and bound antibody was visualized through Enhanced Chemiluminescence by SuperSignal West Pico Chemiluminescent Substrate.

### Ethics statements

In this study we used sixteen sera from healthy infants (12 to 18 months old), thirty sera from healthy adults (21 to 75 years old), and thirty sera from patients (0 to 81 years old) with proven *Staphylococcus aureus* infection as the only microbiological etiology of disease. Sera from *S. aureus* infected patients, collected the day after the hospitalization, were obtained, as well as healthy infants sera, in a collaboration with University Unit of Infectious Diseases (Siena Hospital, Italy); whereas healthy adult sera were purchased from 3H Biomedical AB (Uppsala, Sweden). Written consent was obtained for each serum collected from Siena Hospital. In particular, with regards to healthy infants, written informed consents from the next of kin, caretakers, or guardians on the behalf of the minors/children participants involved in the study were obtained for each single patient. All research involving human participants were approved by the Ethical Committee of the Azienda Ospedaliera Universitaria Senese at Siena Hospital, and were conducted according to the principles expressed in the Declaration of Helsinki. Regarding animal research, this study was carried out in strict accordance with the recommendations enclosed in the Novartis Animal Welfare Policies. The protocol was approved by the Committee on the Ethics of Animal Experiments of Novartis (Permit Number: 201105), and all efforts were made to minimize suffering.

### ELISA

ELISA procedures were performed as already reported [54] with minor modifications. Briefly, Nunc MaxiSorp® flat-bottom 96-well plates (Thermo Scientific) were coated (100 µl per well) overnight at 4°C with 2 µg/ml of CnaBE3 protein in PBS. The plates were washed three times with TPBS and blocked with 200 µl per well of Blocking Buffer containing 3% BSA (Sigma-Aldrich) in PBS for 2 hr at 37°C. Human test sera from patients affected by *Staphylococcus aureus*, from healthy adults, and from healthy infants were initially diluted 1:100 in Dilution Buffer (1% BSA in TPBS) added in duplicate to the wells (100 µl per well), and serially two-fold diluted. After 2 hours incubation at 37°C, the plates were washed three times with TPBS, then Dilution Buffer containing goat anti-human IgG (λ-chain specific) alkaline phosphatase conjugate antibodies (Sigma-Aldrich) diluted 1:2,000 was added 100 µl per well. Following 1hr and 30 min incubation at 37°C, the plates were washed three times with TPBS, and 100 µl per well of a solution of DEA buffer (1M diethanolamine, 0.5 mM MgCl_2_, 0.02% sodium azide, pH 9.8) containing 3 mg/ml p-nitrophenyl phosphate (Sigma-Aldrich) were applied. The reaction was stopped after 20 min by adding 100 µl 4N NaOH. Optical densities (ODs) at 405 nm were measured using SpectraMax 190 Absorbance Microplate Reader supplied with SoftMax® Pro Data Acquisition & Analysis Software (Molecular Devices). To calculate the serum titers and correct experimental variability, two wells per plate received only Dilution Buffer (blank wells), and a standard serum was assayed in parallel with the other sera on each plate. The blank wells were used as negative control and the mean of their OD_405_ nm reading was subtracted from all the sample OD_405_ nm readings. To every two-fold dilution point of the standard serum, made by pooling same volumes of sera highly responsive to CnaBE3, was assigned a value of Arbitrary Units (AU). The first dilution in the linear range of response which gave an OD_405_ nm <= 2.5 was given 1,000 AU, then 500 AU, 250 AU, 125 AU, 62.5 AU from the second to the fifth two-fold dilution point. The standard curve was fitted to a function, which was used to convert the absorbance of sera dilutions into AU [28,55,56]. Sera dilution points with OD_405_ nm >= 0.2 and <= 2.5 were considered in the calculation. Each AU value from each dilution point of a serum was multiplied by the reverse of the corresponding dilution (normalized AU) and the final AU value was calculated as the mean of the normalized AU values of the serum. Every serum was run in duplicate and final AUs were the mean of the AUs of both duplicate samples. Samples that did not fall within an acceptable OD_405_ nm range were re-tested at an alternate dilution. ELISA AU values were natural logarithm transformed to more closely approximate a normal distribution (lnAU). Each experiment was performed in duplicate. The statistical analysis was performed with Mann-Whitney U test.

### In vivo protection assays

Female CD1 5-week old mice were immunized intravenously at days 0 and 14 with a vaccine formulation including 10 µg of each antigen (either the SdrE full length protein, or the CnaBE3 domain) formulated in alum hydroxide (Alum). Negative control groups consisted of mice immunized with adjuvant alone. About 2 weeks after the 2^nd^ immunization, mice were infected intravenously with 100 µl of a bacterial suspension containing 1.5 x 10^7^ CFU of Newman strain or 2.8 x 10^7^ CFU of NCTC8325 strain. Mice were monitored on a daily basis for 4 days after treatment and then euthanized and kidneys collected for bacterial counting. Data were reported as geometric mean and Mann-Whitney U-test was used as statistical analysis.

### Opsonophagocytic killing assay

Human promyelocytic leukemia cells HL-60 (ATCC CCL240) were maintained in enriched medium and differentiated into phagocytes using 0.8% N,N-Dimethylformamide (DMF, Sigma). Following heat inactivation (30 min, 56°C), mouse SdrE and CnaBE3 antisera were prediluted 1:50 in Hank’s Balanced Salt Solution (HBSS) buffer (with Ca^2+^/Mg^2+^). Newman strain grown overnight in TSB 2% NaCl_2_, was washed once in PBS, and then incubated with serum (75,000 CFU/well) at 4°C for 20 minutes. Differentiated HL-60 cells were distributed at 3.7 × 10^6^ per well (HL-60: bacteria ratio, 50:1) and rabbit complement was added at 10% final concentration. At this point, samples were diluted in H_2_O + tween 0.05% in order to completely lyse HL-60 cells, and plated onto TSA plates to enumerate the number of colonies (Time 0). Contemporary, a replica of the same samples was incubated at 37°C for 1 hour, under agitation at 600 rpm. The reaction was stopped by putting the plate on ice, and samples were diluted in H_2_O + tween 0.05% and plated for CFU count determination (Time 1). The killing percentage was calculated using the following formula: [(Time 0 – Time 1)/Time 0] * 100, where Time 0 is the number of colonies before the incubation and Time 1 is the number of colonies after 1 hour of incubation.

### Differential scanning fluorimetry

DSF experiments were performed using thin wall PCR plates (Axigen). SdrE was used at a final concentration of 10 µM; both CaCl_2_ (dissolved in water) and EDTA, were used at up to 1mM. The SYPRO orange dye 5000X (Invitrogen) was used at the final concentration of 5X in each well. The reaction mixtures were 40 µl in 50 mM Tris-HCl buffer, pH 7.6. Fluorescence intensities were monitored using an Mx3005 RT-PCR instrument (Stratagene) using the FAM (492 nm) and ROX (610nm) filters for excitation and emission, respectively. Samples were heated from 25°C to 95°C at a 1 °C/min scan rate. The melting temperature (Tm) values were extrapolated by fitting the raw data to a Boltzmann model using GraphPad Prism 5.0 software. Each experiment was performed at least in triplicate.

### Proteolysis Assay

SdrE protein, CnaBE3 domain and CnaBE3 domain short form were dialyzed overnight either in 50mM Tris HCl, 1mM CaCl_2,_ pH 7.6 or in 50mM Tris HCl, 1mM EDTA, with one change of buffer. Dialyzed proteins were treated with 0.1% RapiGest™ (Waters), boiled at 100°C for 10 min. Samples were allowed to cool and were digested with sequencing grade modified trypsin (Promega), using an enzyme/substrate ratio of 1:25. The reaction was carried out at 37°C. After 24h of incubation, 15 µl were drawn out and mixed with boiling SDS sample buffer. SDS-PAGE analysis was performed using NuPage 4-12% Bis-Tris gradient gels (Invitrogen) according to the manufacturer’s instructions followed by Coomassie Blue staining.

### Ultra Performance Liquid Chromatography Size-Exclusion Chromatography (UPLC-SEC) purification of the 37kDa trypsin resistant fragment

The 37kDa resistant fragment was purified by Ultraperformance liquid chromatography SEC. The overnight trypsin digested SdrE sample was purified using Bridged Ethyl Hybrid 200 (BEH200) column in UPLC (Waters) at 0.5 ml/min with 20 mM phosphate buffer, pH 8.0. The overnight trypsin digested SdrE was loaded onto the column, peaks were assigned at A_280_, and apparent molecular masses were determined from a standard curve. The purified polypeptide identified from the collected fractions was successively used for N-terminal sequencing by Edman Degradation.

### Mass spectrometry analyses

As previously reported [57], spots of colloidal Coomassie blue stained bands were excised from the SDS-PAGE gel using a Pasteur pipette and destained overnight in 200 µl of 50% acetonitrile, 50 mM ammonium bicarbonate. The spots were then washed with 200 µl of acetonitrile. The acetonitrile was discarded, and the spots were allowed to air dry. Modified trypsin (12 µg/ml) in 5 mM ammonium bicarbonate was added to each spot, and the enzymatic digestion was performed overnight at 37°C. 0.8 µl of the digestion was directly spotted on a PAC (Prespotted AnchorChip 96 set for proteomics; Bruker Daltonics) target. The air dried spots were washed with 0.6 µl of a solution of 70% ethanol, 0.1% trifluoroacetic acid (TFA). Peptide mass spectra were recorded with a matrix-assisted laser desorption ionization-time of flight MALDI-TOF/TOF mass spectrometer (UltraFlex; Bruker Daltonics, Bremen, Germany). Ions generated by laser desorption at 337 nm (N_2_ laser) were recorded at an acceleration of 25 kV in the reflector mode. About 200 single spectra were accumulated for improving the signal/noise ratio and analyzed by FlexAnalysis (version 2.4; Bruker Daltonics). Peptide identiﬁcation was performed using BioTools and Sequence Editor 3.0 (Bruker Daltonics). Initial searches against SdrE sequence using Mascot search engine version 2.0.05 (Matrix Science) identified the peptides from the resistant fragment in the CnaB region of the protein only.

### Amino-terminal sequencing

To confirm the identity of the purified overnight trypsin major resistant fragment of SdrE, the UPLC SEC purified resistant fragment of SdrE was subjected to amino-terminal sequence analyses by Edman degradation [58]. Amino-terminal sequence analyses were performed on an Agilent G1000A series protein sequencer following manufacturer’s protocol. 

## Supporting Information

Figure S1 **Ca^2+^ stabilizes SdrE protein.** SdrE protein was tested for its capacity to bind calcium in Differential Scanning Fluorimetry (DSF) experiments. SdrE protein was incubated in absence of Ca^2+^ ions or in presence of either 1mM CaCl_2_ or 1mM of EDTA. In presence of 1 mM CaCl_2_ the melting temperature (Tm) of SdrE protein increased up to 54.43°C, whereas after the incubation with EDTA the Tm decreased to 39.72°C, below the standard Tm of SdrE protein that was equal to 45.26°C. These data indicate that calcium binds to SdrE protein, favoring the structural stability of the protein.
(TIF)

Figure S2 **

*Aminoacid* sequence comparison between B3 repeat of the SdrE protein and subdomain D1 of the CnaB1 domain of the *S. aureus* protein Cna.** A) Sequences alignment between B3 repeat of the SdrE protein (BE3) and subdomain D1 of the CnaB1 domain of the Cna protein. Identical residues are highlighted and the hypothesized new (green box) and current (light red box) putative CnaB domains are shown. B) Amino acid sequences of B repeats of the Sdr proteins are shown. The putative CnaB domains so far reported are encompassed in a light red box, whereas a green box highlights the suggested new CnaB domain sequences. Moreover a light blue box delimits a consensus Ca^2+^ binding EF-hand loop, present in all the B repeats of the Sdr proteins.
(TIF)
