# Supplementary figures and images for: Protective Activity of the CnaBE3 Domain Conserved among Staphylococcus aureus Sdr Proteins
Source: PLoS One. 2013 Sep 17;8(9):e74718. doi: 10.1371/journal.pone.0074718 (PMC3775735; doi:10.1371/journal.pone.0074718)

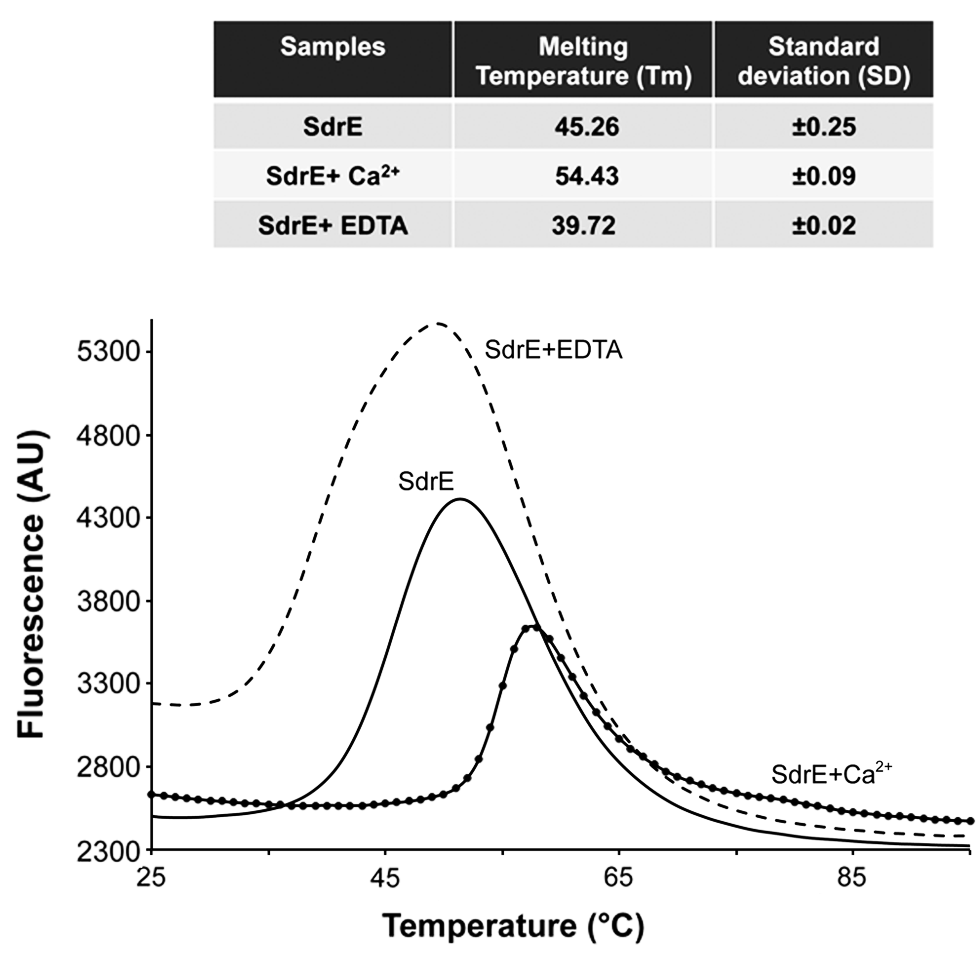

Supplement: Figure S1 — Ca2+ stabilizes SdrE protein. SdrE protein was tested for its capacity to bind calcium in Differential Scanning Fluorimetry (DSF) experiments. SdrE protein was incubated in absence of Ca2+ ions or in presence of either 1mM CaCl2 or 1mM of EDTA. In presence of 1 mM CaCl2 the melting temperature (Tm) of SdrE protein increased up to 54.43°C, whereas after the incubation with EDTA the Tm decreased to 39.72°C, below the standard Tm of SdrE protein that was equal to 45.26°C. These data indicate that calcium binds to SdrE protein, favoring the structural stability of the protein. (TIF) [file pone.0074718.s001.tif]

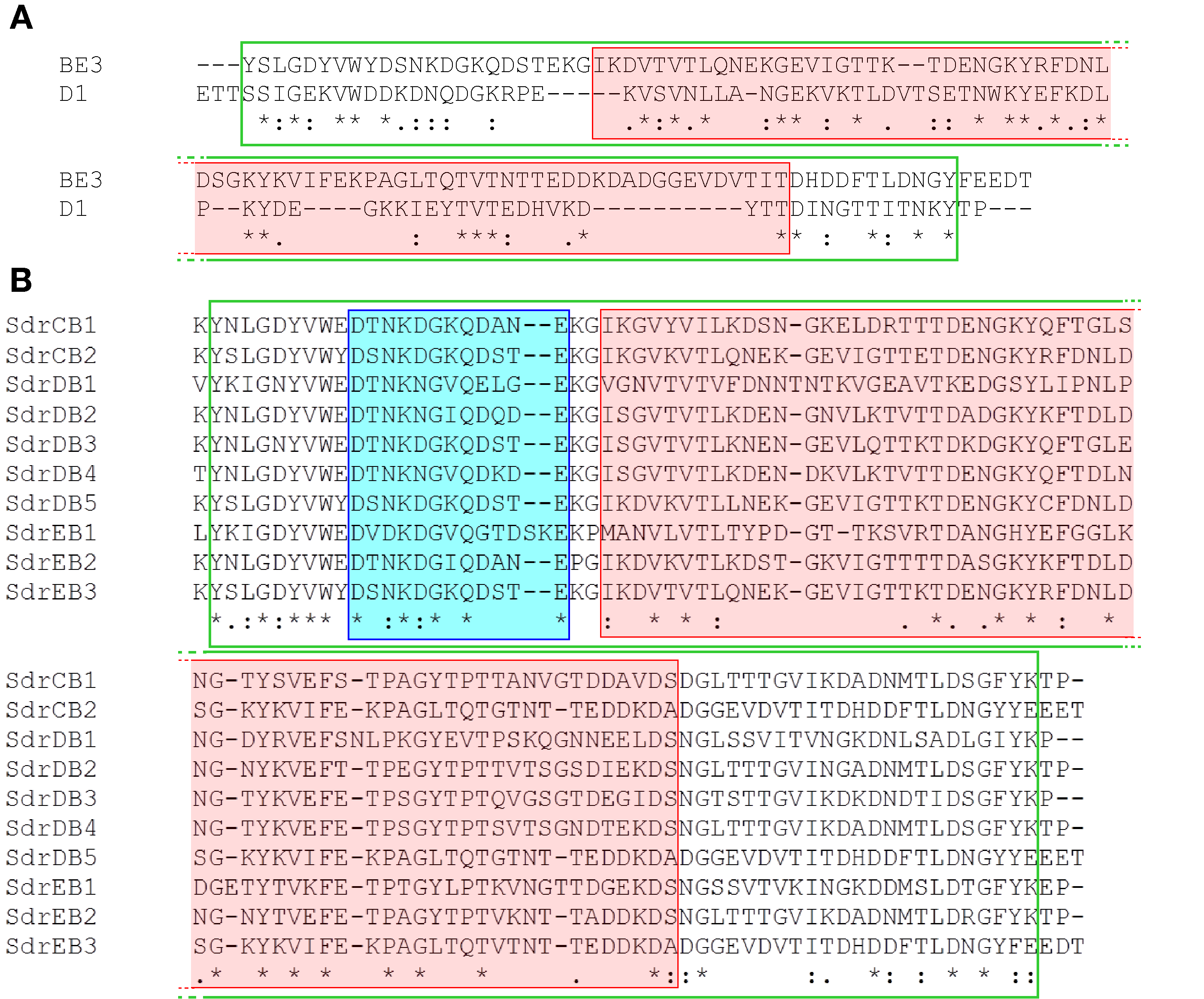

Supplement: Figure S2 — Aminoacid sequence comparison between B3 repeat of the SdrE protein and subdomain D1 of the CnaB1 domain of the S. aureus protein Cna. A) Sequences alignment between B3 repeat of the SdrE protein (BE3) and subdomain D1 of the CnaB1 domain of the Cna protein. Identical residues are highlighted and the hypothesized new (green box) and current (light red box) putative CnaB domains are shown. B) Amino acid sequences of B repeats of the Sdr proteins are shown. The putative CnaB domains so far reported are encompassed in a light red box, whereas a green box highlights the suggested new CnaB domain sequences. Moreover a light blue box delimits a consensus Ca2+ binding EF-hand loop, present in all the B repeats of the Sdr proteins. (TIF) [file pone.0074718.s002.tif]
